# Long‐term outcomes of nationwide coordinated neovascular AMD treatment: A study based on Polish national registry

**DOI:** 10.1111/aos.70040

**Published:** 2025-11-17

**Authors:** Sławomir Teper, Daniel Ledwoń, Adam Sendecki, Patrycja Romaniszyn‐Kania, Aleksandra Tuszy, Julia Nycz, Andrzej W. Mitas, Małgorzata Figurska, Edward Wylęgała, Marek Rękas

**Affiliations:** ^1^ Chair and Clinical Department of Ophthalmology, Faculty of Medical Sciences in Zabrze Medical University of Silesia Katowice Poland; ^2^ Scientific Research Facility, Branch in Bielsko‐Biala Medical University of Silesia Katowice Poland; ^3^ Faculty of Biomedical Engineering Silesian University of Technology Zabrze Poland; ^4^ Institute of Biomedical Engineering and Informatics Technische Universität Ilmenau Ilmenau Germany; ^5^ Department of Ophthalmology, Military Institute of Medicine Central Clinical Hospital of the Ministry of National Defense Warsaw Poland; ^6^ National Consultant in Ophthalmology, Ministry of Health Warsaw Poland

**Keywords:** anti‐VEGF therapy, long‐term outcomes, national registry, neovascular AMD, real‐world evidence

## Abstract

**Purpose:**

To evaluate long‐term outcomes of anti‐VEGF therapy for nAMD using the Polish Retinal Therapeutic Program Monitoring Registry.

**Methods:**

The cohort consisted of 63 840 eyes with nAMD qualified for treatment from 2016 to 2022, with follow‐up through 31 October 2023. Long‐term follow‐up (>5 years) was conducted for 7631 eyes. Statistical comparisons and multivariate logistic regression analysed differences between treatment failure and long‐term treatment, treatment effectiveness, drug efficacy and predictive factors.

**Results:**

The relative risk of nAMD in females was 1.45. By the last follow‐up, 17 518 (48.6%) patients discontinued treatment due to patient‐related factors, 7202 (20.0%) due to treatment failure, 6285 (17.4%) for other reasons and 5065 (14.0%) due to death or clinical complications. Long‐term patients were significantly younger, more of them were previously treated, received fewer injections per year and had better baseline BCVA and lower CRT than the failure group. Females and previously treated patients had a higher likelihood of long‐term therapy, while older age and initial ranibizumab treatment increased the risk of failure. Patients with an initial BCVA >0.4 logMAR had significantly worse BCVA outcomes, while CRT and treatment strategy showed no significant differences. Introduction of programme exclusion if treatment gap >4 months reduced long breaks, increased injection numbers, slightly lowered BCVA and had no significant effect on CRT.

**Conclusion:**

Early visual acuity response, drug choice (aflibercept), and healthcare system modifications influence long‐term success and adherence in nAMD management. While systemic changes improved continuity and injection frequency, they did not enhance visual outcomes, highlighting the need for individualized treatment strategies.

## 1 INTRODUCTION

The introduction of intravitreal anti‐vascular endothelial growth factor (anti‐VEGF) therapies has revolutionized the management of neovascular age‐related macular degeneration (nAMD), significantly improving visual outcomes compared to previous treatment modalities (Rosenfeld et al., 2006). Clinical trials have demonstrated the efficacy of agents such as ranibizumab, aflibercept and bevacizumab in stabilizing or improving vision in patients with nAMD (CATT Research Group et al., 2011). However, outcomes observed in randomized controlled trials may not fully reflect real‐world effectiveness due to strict inclusion criteria and controlled settings (Chakravarthy et al., 2013).

Understanding long‐term real‐world outcomes of nAMD treatments is essential for clinicians and policymakers to optimize patient care and resource allocation. National registries offer a valuable tool for collecting large‐scale, longitudinal data in routine clinical practice, providing insights into treatment effectiveness, safety and adherence over extended periods (Black, 1997). In ophthalmology, registries such as the Fight Retinal Blindness! Registry (Gillies et al., 2014), the Swedish Macula Register (Westborg et al., 2017) and the American Academy of Ophthalmology's IRIS Registry (Tomaiuolo et al., 2023) have contributed significantly to our understanding of real‐world treatment outcomes in nAMD. Similarly, in other medical specialties, national registries have been instrumental in advancing patient care by facilitating epidemiological studies, monitoring treatment patterns and evaluating long‐term outcomes (Heloterä et al., 2024; Purola et al., 2025; Subhi et al., 2024). In Poland, the establishment of a national registry for nAMD provides an opportunity to analyse real‐life treatment outcomes in a large patient cohort over an extended follow‐up period (Figurska et al., 2020; Teper et al., 2022, 2025).

This study aims to assess the long‐term outcomes of anti‐VEGF therapy for nAMD using data from the Retinal Therapeutic Program Monitoring Registry (RTPMR), a part of the Polish Therapeutic Program Monitoring System. By evaluating visual acuity changes, treatment patterns and factors influencing outcomes in a real‐world setting, we seek to contribute valuable information that may enhance clinical practice and inform health policy decisions in ophthalmology.

## 2 MATERIALS AND METHODS

### 2.1 Dataset

This non‐randomized, retrospective, observational, multicentre study used data from RTPMR collected from 1 January 2016 to 31 October 2023. The observed patients were qualified between January 2016 and 31 December 2022. During this period, the RTPMR Coordination Committee (CC) processed 83 530 requests for qualification, of which it rejected requests for 13 873 (16.6%) patients. The dataset includes 69 515 treatment episodes for 63 840 eyes of 55 011 patients, accounting for requalification after prior treatment within the RTPMR.

The data stored in the RTPMR are anonymized and de‐identified; therefore, institutional review board approval and written informed consent were not required for this study. The research was conducted in accordance with the principles of the Declaration of Helsinki.

The RTPMR applied the following eligibility criteria: (1) presence of active macular neovascularization (MNV) occupying more than 50% of the AMD‐related changes; (2) age over 45 years; (3) total degenerative lesion size of less than 12 optic disc areas; (4) best‐corrected visual acuity (BCVA) between 0.2 and 0.8 on the decimal scale, as determined using the Snellen chart or its ETDRS equivalent (34–80 letters); (5) patient consent for intravitreal injections; and (6) absence of dominant geographic atrophy, haemorrhage or significant permanent damage to the foveal structure, such as fibrosis, foveal atrophy or a significant chronic scar.

The dataset included each patient's age, gender, affected eye and date of diagnosis, as well as the following information from each control visit (with and without intravitreal injection): visit date, an OCT file, optionally other imaging tests, central retinal thickness (CRT), BCVA according to the Snellen or ETDRS chart, percentage of the area occupied by neovascular lesions relative to the total AMD‐affected area, presence of haemorrhage, atrophy or scarring, and details of drug administration, including the drug name. The BCVA measures were converted to their logMAR equivalents.

### 2.2 Research questions

The following research questions were formulated to assess long‐term outcomes:

1. What are the predictive factors in the study population?
2. Why do patients stop treatment?
3. What is the difference between patients who do not continue treatment and those who have been treated for more than 5 years?
4. What is the effectiveness of treatment over at least 5 years of follow‐up?
5. Does long‐term treatment with ranibizumab and aflibercept differ in effectiveness, injection intervals, percentage of people discontinuing treatment before 5 years and for what reason?
6. How changes in inclusion and exclusion criteria affected treatment?

### 2.3 Outcome measures

To evaluate treatment outcomes, we first compared the sex and age characteristics of patients enrolled in the RTPMR with demographic data from the Polish population aged 45 years and older in 2023. This comparison allowed us to estimate the total treatment needs and identify relative risk factors.

Based on treatment duration and patient exclusions, we identified two groups for further comparison: eyes treated in the RTPMR for at least 5 years (long‐term group) and eyes with treatment discontinued earlier due to disease progression or permanent foveal damage (treatment failure group). Comparisons included patient age at treatment initiation, sex, BCVA and CRT (at baseline, after the third injection, at the last measurement and as a mean value over the entire treatment course), number of eyes treated before entering the RTPMR (new or continued therapy), first medication used (aflibercept or ranibizumab) and annual injection rate.

To identify predictive factors, we conducted a logistic regression analysis, differentiating between the long‐term treatment and treatment failure groups based on initial treatment patterns. Independent variables included sex, age, therapy type (new or continued), medication administered, BCVA and CRT after each injection and the time intervals between the first and second as well as the second and third injections.

Treatment outcomes in the long‐term group were presented using quantitative summaries and comparative analyses based on grouping variables. These comparisons included patient age at treatment initiation, sex, BCVA and CRT (initial, after the third injection, last measured and mean values), number of eyes treated before RTPMR enrolment (new or continued therapy), drug switching, first medication and annual injection rate. Additionally, BCVA trends over successive treatment years were illustrated using bar charts, showing the percentage of eyes with BCVA ≤0.4 logMAR, categorized by different grouping variables.

### 2.4 Statistical analysis

For comparisons between two independent groups, an independent *t*‐test was used when normality (Shapiro–Wilk test) and homogeneity of variances (Levene's test) assumptions were met. Given the sample size, for some continuous variables (e.g. age), the *t*‐test was used based on the central limit theorem, following graphical distribution assessment (Q‐Q plot). Effect size was measured using Cohen's d, and a 95% confidence interval (CI) for the mean difference was estimated using the *t*‐distribution. If normality was violated, the Mann–Whitney *U* test was applied, with rank‐biserial correlation (rg) as the effect size measure. The median difference and its CI were estimated using the Hodges–Lehmann estimator. Comparisons of more than two independent groups were conducted using ANOVA when assumptions of normality and homogeneity of variance were met, with effect size reported as eta squared (*η* ^2^). Tukey's HSD test was used for post hoc pairwise comparisons. If assumptions were violated, the Kruskal–Wallis test was applied, with epsilon squared (*ε* ^2^) as the effect size measure, followed by Dunn's post hoc test with Bonferroni correction. Repeated measures analysis was conducted to compare outcome measures at three treatment stages (initial, after the third injection and last observation). When assumptions were met, repeated measures ANOVA with Tukey's HSD post hoc test was used; otherwise, the Friedman test was applied, with Kendall's *W* as the effect size measure. Post hoc comparisons were performed using the Wilcoxon signed‐rank test with Bonferroni correction, and effect size was reported as Wilcoxon's *r*. The median difference and its CI were estimated via bootstrapping. Categorical variable comparisons were conducted using the chi‐square test, with effect size reported as Cramér's *V*. Relative risk (RR) and its CI were estimated using contingency tables. A multivariate logistic regression model was employed to assess the influence of independent variables on the likelihood of long‐term treatment compared to treatment failure.

## 3 RESULTS

**FIGURE 1:**
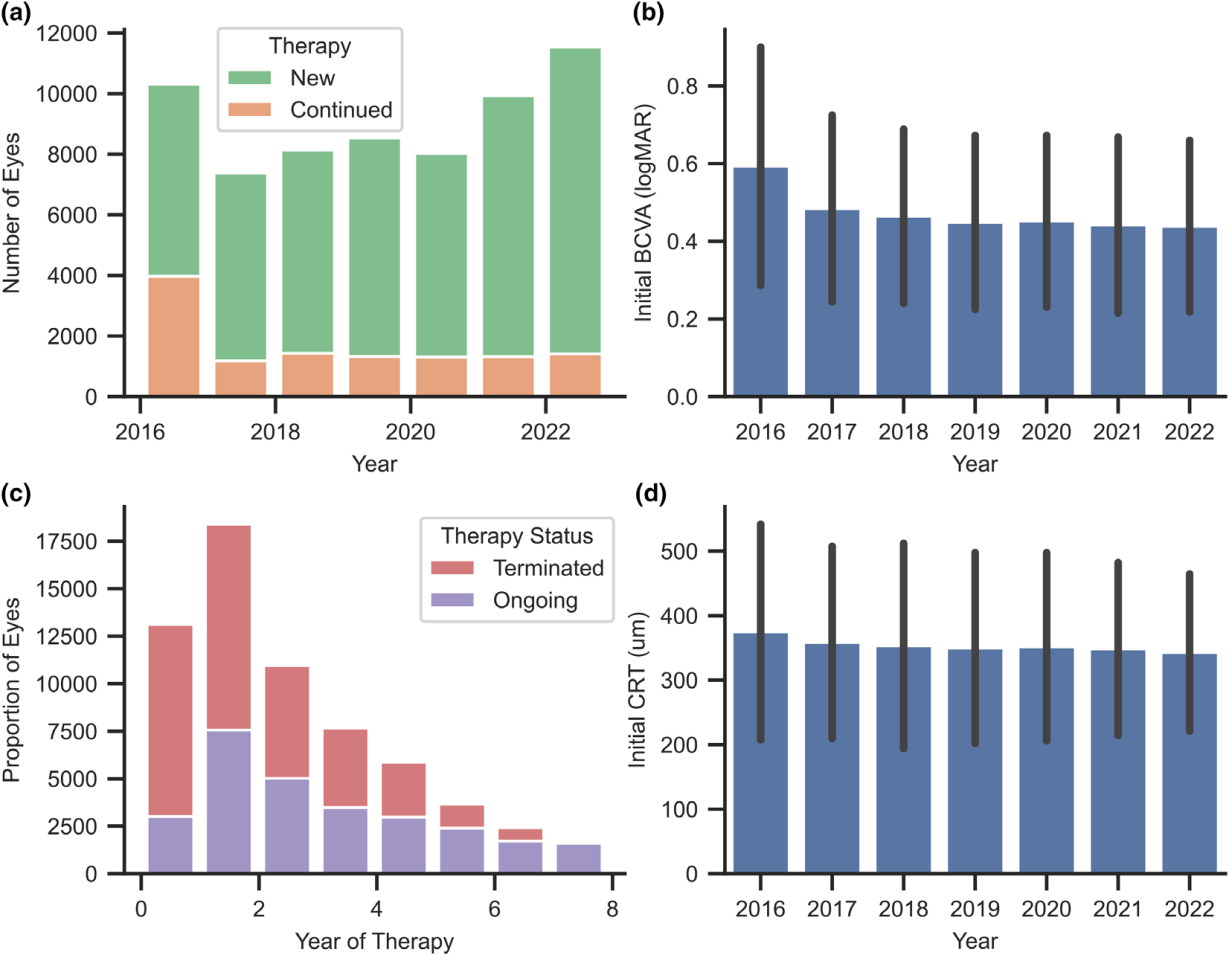
Summary of the number of eyes enrolled in the Retinal Therapeutic Program Monitoring Registry in consecutive years by new and continuing treatments (a) along with the mean and standard deviation of BCVA (b) and CRT (d) and the proportion of terminations after consecutive years of treatment (c).

Of all 63 840 eyes participating in the RTPMR, 51 902 (81.3%) were treatment‐naïve, and the remaining 11 938 (18.7%) had been previously treated. The majority of continued therapies began in 2016, the year the RTPMR started collecting data. The number remained similar at around 1300 eyes each year in subsequent years (Figure 1a). The proportion of terminations remains similar in the first years of treatment, while for therapies lasting a minimum of 5 years it decreases significantly relative to ongoings (Figure 1c). Initial BCVA (Figure 1b) and CRT (Figure 1d) remain at similar levels in subsequent years of the treatment course. The higher BCVA in 2016 is due to less restrictive criteria for patients to be included in the treatment, which were later revised.

Of the 55 011 patients, 46 182 (84%) had unilateral disease, while the remaining 8829 (16%) received treatment in both eyes. The 5561 (63%) treatment‐naïve patients initiated therapy in both eyes, and 966 (11%) continued prior treatment. In the remaining 2302 (26%) patients, therapy for the first eye continues the previous treatment, with the second eye enrolled as treatment‐naïve. The interval between the start of therapy for the first and second eye varied across the 7‐year follow‐up period, with 2272 (25.73%) enrolments of both eyes co‐occurring at the first visit and 4489 (50.8%) enrolments of the second eye in the first year after the start of therapy.

Table 1 shows the sex and age characteristics of all patients with nAMD participating in the RTPMR throughout the observed period and compares patient characteristics in 2023 with population data limited to those over 45. The total treatment needs of nAMD in the population over 45 years of age is 0.2315% (95% CI: 0.2313%–0.2316%). The age of patients with nAMD is significantly higher than that of the general population. The relative risk of nAMD for females is significantly higher compared to males. Considering the age at which treatment starts, this relationship is found in the 55–64 and 65–74 age ranges.

**TABLE 1:** Characteristics of the patients enrolled in the Retinal Therapeutic Program Monitoring Registry between 1 January 2016 and 13 October 2023 and patients in 2023 compared to the Poland population characteristics in 2023.

Characteristic	All patients with nAMD in RTPMR (*n* = 63 840)	Patients with nAMD in RTPMR in 2023 (*n* = 40 347)	Poland population in 2023 aged over 45 (*n* = 17 432 143)	Effect size	*p* value
Age, years	75.89 ± 8.21	75.43 ± 8.15	62.61 ± 11.85	MD = 12.82 (12.74–12.9)	<0.001^a^ d = 1.26
Sex, *n* (%)				RR = 1.45 (1.42, 1.48)	<0.001^b^
Female	40 212 (62.99)	25 688 (63.67)	9 541 625 (54.74)		
Male	23 628 (37.01)	14 659 (36.33)	7 890 518 (45.26)		
Age groups, *n* (%)					
45–54 years				RR = 0.94 (0.79, 1.12)	0.53^b^
Female	393 (50.32)	245 (48.61)	2 910 727 (50.10)		
Male	388 (49.68)	259 (51.39)	2 898 528 (49.90)		
55–64 years				RR = 1.24 (1.17, 1.32)	<0.001^b^
Female	3400 (56.39)	2297 (57.71)	2 406 674 (52.34)		
Male	2629 (43.61)	1683 (42.29)	2 191 561 (47.66)		
65–74 years				RR = 1.20 (1.16, 1.24)	<0.001^b^
Female	13 795 (60.37)	9582 (61.08)	2 529 340 (56.73)		
Male	9055 (39.63)	6106 (38.92)	1 929 298 (43.27)		
75+ years				RR = 1.05 (1.02, 1.09)	<0.001^b^
Female	22 624 (66.19)	13 564 (67.23)	1 694 884 (66.05)		
Male	11 556 (33.81)	6611 (32.77)	871 131 (33.95)		

Abbreviations: D, Cohen's d; MD, mean difference; nAMD, neovascular age‐related macular degeneration; RR, relative risk; RTPMR, Retinal Therapeutic Program Monitoring Registry.

^a^ Welch *t*‐test.

^b^ Chi‐square test.

At the last observed time point, therapies for 36 070 eyes had been terminated, and the remaining 27 770 are ongoing. The largest number of terminations, at 17518 (48.6%), is due to patient‐related factors associated with a lack of active participation in treatment (Table 2). Patient death and other causes are further common factors for treatment termination. The mean age at death as a cause of termination is 80.3 ± 7.2 for women and 78.8 ± 7.5 for men, with a life expectancy for the Polish population of 82 years for women and 74.7 for men. In 7202 (20.0%) cases, treatment failed, resulting in disease progression and the development of permanent damage. The remaining least frequent causes of termination relate to clinical complications and account for 1096 (3.0%) cases. Of these, 85 (0.13% of all subjects; 0.009% of all injections) terminations occurred due to active severe endophthalmitis.

**TABLE 2:** The reasons for treatment course terminations and the number of eyes in 7 years of follow‐up in the Retinal Therapeutic Program Monitoring Registry.

Reason for treatment termination	Number of eyes
Patient‐related factors	
No active treatment within 4 months	12 267
Patient's withdrawal from treatment	3597
Lack of cooperation between the patient and the attending physician	1654
Other	6285
Death	3969
Treatment failure	
Disease progression	3227
Presence of permanent damage to the fovea structure	
Other permanent lesions	1206
Fibrosis	1143
Atrophy	899
Significant chronic disciform scar	727
Medical or clinical complications	
Adverse reactions associated with the active substance	710
Hypersensitivity to the active substance	139
Tractional retinal detachment or a full thickness macular hole	107
Active severe endophthalmitis	85
Active infection of the eye or its vicinity	55

**FIGURE 2:**
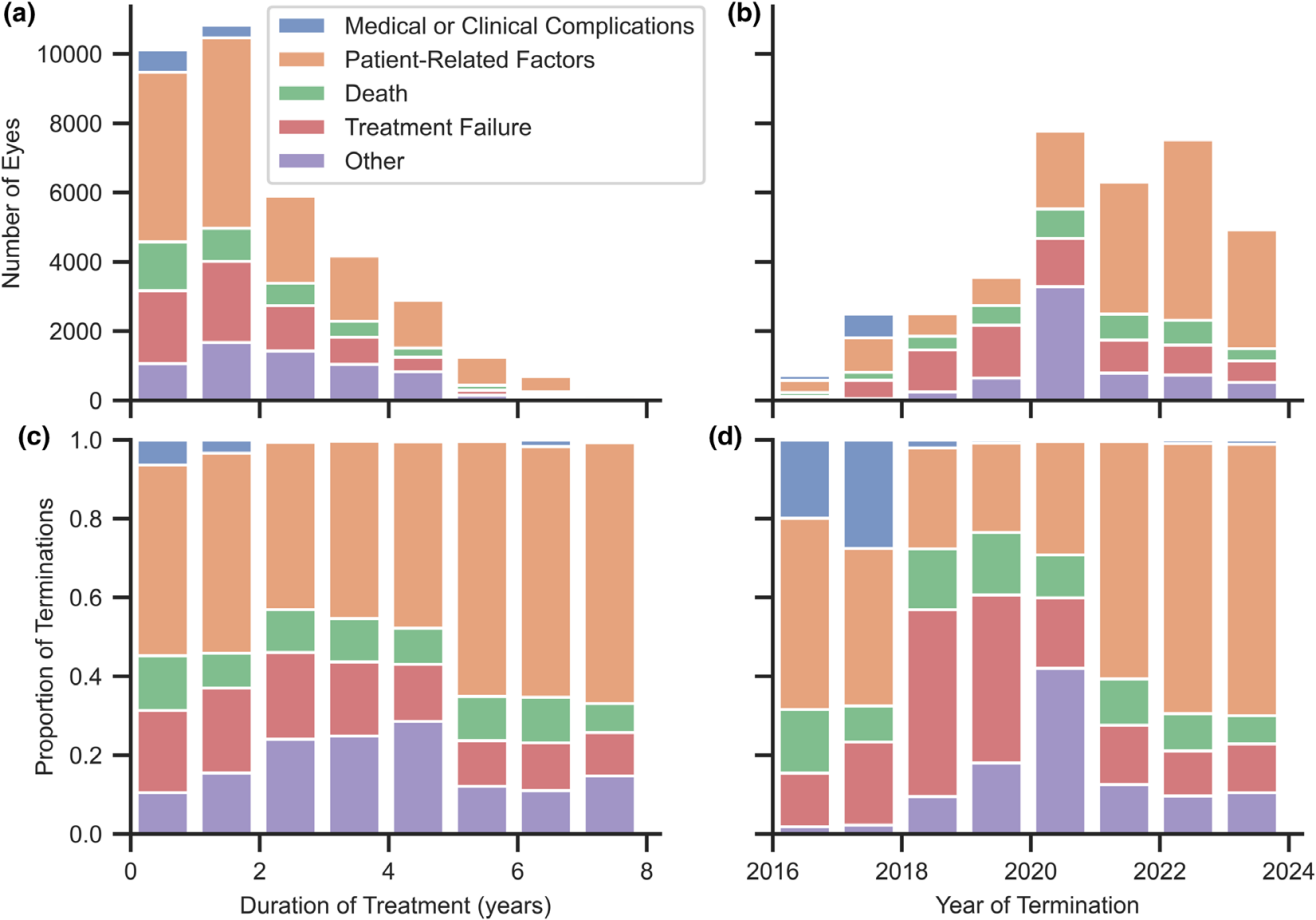
Summaries of the contribution of each reason for treatment termination in subsequent years.

The incidence of each reason for treatment termination in successive years of the RTPMR and the duration of therapy is shown in Figure 2. The highest number of terminations occurred in the first 2 years after starting treatment, with patient‐related factors dominating in subsequent years. The absolute decrease in terminations in subsequent years of treatment is also due to the lack of sufficiently long follow‐up for patients included in the RTPMR in recent years. Proportionally, the contribution of individual causes relative to treatment time has remained similar. Only a slight increase in the relative share of other causes and a decrease in the relative share of treatment failure in the first 5 years of treatment can be observed.

We compared the characteristics and outcomes in the group of 7631 patients on long‐term treatment to the 6969 patients from the treatment failure group. Patients on long‐term treatment were significantly younger at baseline than patients in the failure group, with no significant differences in sex (Table 3). Most of the therapies were new, with slightly more continuation in therapies in long‐term‐treated patients. In both groups, the ratio of initial medicines selected for the patient treatment is similar. The average number of injections per year in patients on long‐term treatment is slightly lower, which may be due to the lower number of injections in the following years. Patients in the failed treatment group started therapy with significantly worse BCVA and slightly higher CRT.

**TABLE 3:** Comparison of characteristics and outcome measures between patients with treatment failure and patients treated long‐term in the Retinal Therapeutic Program Monitoring Registry.

Characteristic	Treatment failure (*n* = 6969)	Long‐term treatment (*n* = 7631)	Effect size, value (95% CI)	*p* value
Age, years, mean ± std	77.14 ± 7.69	72.92 ± 7.79	MD = 4.22 (3.97, 4.47)	<0.001^a^, d = 0.54
Sex, *n* (%)			RR = 0.95 (0.90, 1.00)	0.1275^b^, *V* = 0.01
Female	4370 (62.7)	4879 (63.9)		
Male	2599 (37.3)	2752 (36.1)		
BCVA, logMAR, median (quartile deviation)				
Initial	0.70 (0.10)	0.40 (0.24)	HL = 0.20 (0.18, 0.28)	<0.001^c^, rg = −0.42
After third injection	0.70 (0.15)	0.30 (0.15)	HL = 0.20 (0.18, 0.25)	<0.001^c^, rg = −0.47
Last measured	0.90 (0.15)	0.40 (0.20)	HL = 0.50 (0.40, 0.55)	<0.001^c^, rg = −0.74
Mean	0.68 (0.12)	0.35 (0.12)	HL = 0.31 (0.27, 0.34)	<0.001^c^, rg = −0.75
CRT, μm, median (quartile deviation)				
Initial	322.0 (59.5)	309.0 (53.0)	HL = 13.0 (−18.0, 43.0)	<0.001^c^, rg = −0.08
After third injection	274.0 (53.0)	277.0 (49.5)	HL = −2.0 (−38.0, 34.0)	0.1214^c^, rg = 0.01
Last measured	268.0 (57.5)	248.0 (42.5)	HL = 20.0 (3.0, 36.0)	<0.001^c^, rg = −0.14
Mean	279.7 (44.2)	265.9 (34.2)	HL = 12.5 (0.1, 25.1)	<0.001^c^, rg = −0.12
Therapy, *n* (%)			RR = 1.57 (1.48, 1.66)	<0.001^b^, *V* = 0.09
New	5520 (79.2)	5407 (70.9)		
Continued	1449 (20.8)	2224 (29.1)		
First medication, *n* (%)			RR = 0.75 (0.71, 0.79)	<0.001^b^, *V* = 0.06
Aflibercept	5099 (73.8)	6026 (79.0)		
Ranibizumab	1821 (26.3)	1605 (21.0)		
Number of injections per year, *n*, median (quartile deviation)	5.95 (1.37)	5.43 (1.07)	HL = 0.48 (0.13, 0.83)	<0.001^c^, rg = −0.14

Abbreviations: BCVA, best corrected visual acuity; CRT, central retinal thickness; D, Cohen's d; HL, Hodges–Lehmann estimator of median difference; MD, mean difference; rg, rank‐biserial correlation; RR, relative risk; *V*, Cramer's *V*.

^a^ Welch *t*‐test.

^b^ Chi‐square test.

^c^ Mann–Whitney *U* test.

**FIGURE 3:**
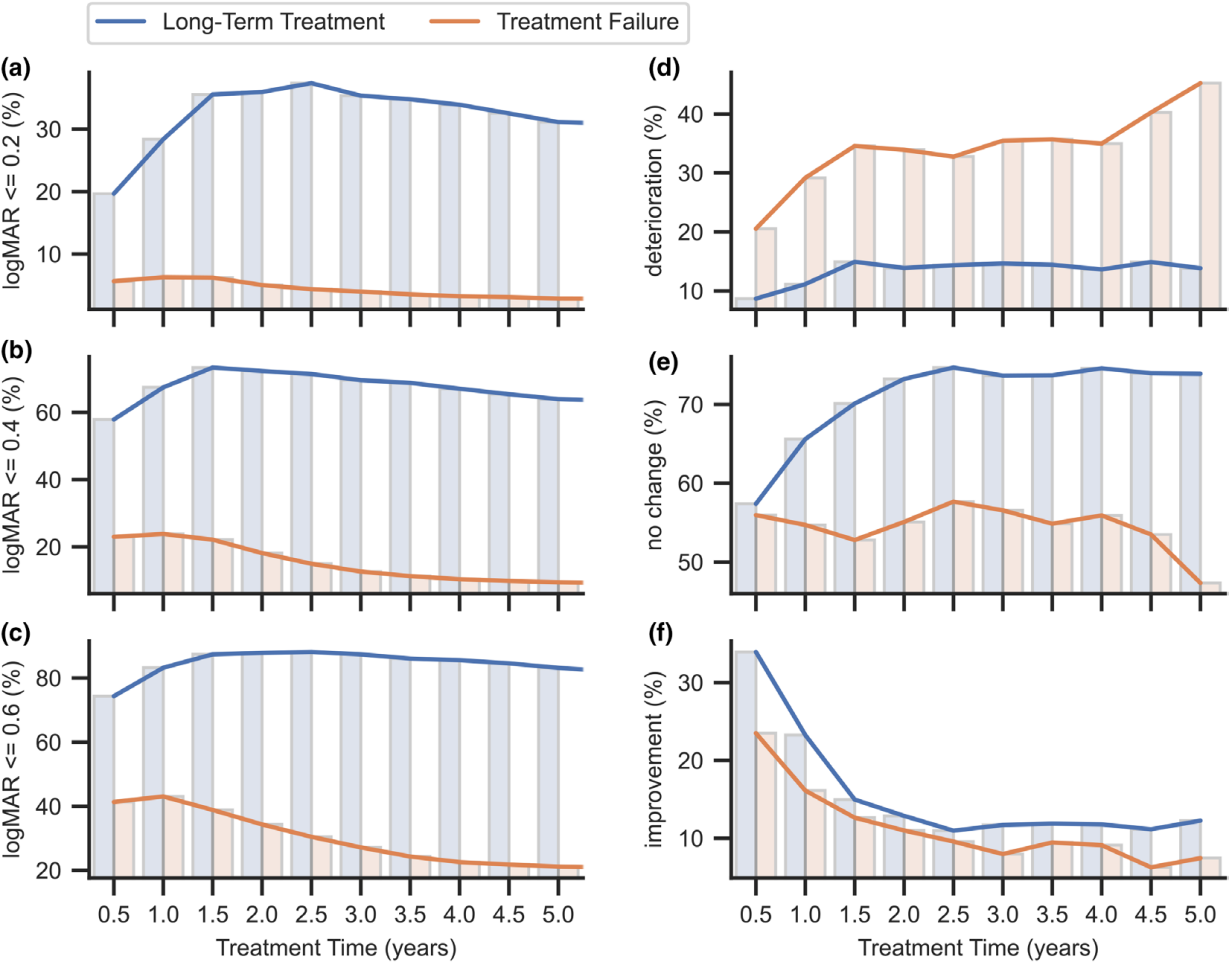
Percentage summaries of the number of eyes with BCVA below established thresholds (a–c) and the trend of BCVA changes in subsequent years of treatment (d–f), divided into failed and long‐term treatments.

The treatment resulted in a significant reduction in retinal thickness in both groups (Table 4). However, visual acuity improved significantly only in the long‐term treatment group, mostly during the early stages of treatment. Conversely, in the treatment failure group, the first three injections had no effect on BCVA, while significant deterioration continued until the last measurement. The percentage contribution in each acuity range and the effectiveness of the therapy over time are presented in Figure 3.

**TABLE 4:** Comparison of visual acuity and central retina thickness at successive stages of therapy of patients among those with treatment failure and those treated long‐term.

Characteristics	Initial versus after third injection	After third injection versus last measured	Initial versus last measured
BCVA, logMAR			
Treatment failure	*p* < 0.001, *r* = 0.11, DM = 0.0 (0.0, 0.0)	*p* < 0.001, *r* = 0.65, DM = 0.27 (0.25, 0.29)	*p* < 0.001, *r* = 0.59, DM = 0.25 (0.25, 0.26)
Long‐term treatment	*p* < 0.001, *r* = 0.381, DM = −0.06 (−0.06, −0.055)	*p* < 0.001, *r* = 0.13, DM = 0.04 (0.035, 0.045)	*p* < 0.001, *r* = 0.14, DM = −0.045 (−0.05, −0.025)
CRT, μm			
Treatment failure	*p* < 0.001, *r* = 0.53, DM = −46.0 (−48.5, −43.5)	*p* < 0.001, *r* = 0.16, DM = −11.0 (−12.5, −9.0)	*p* < 0.001, *r* = 0.54, DM = −57.0 (−59.5, −54.5)
Long‐term treatment	*p* < 0.001, *r* = 0.44, DM = −30.5 (−32.0, −29.0)	*p* < 0.001, *r* = 0.36, DM = −29.0 (−31.0, −27.5)	*p* < 0.001, *r* = 0.59, DM = −60.5 (−62.5, −58.5)

Abbreviations: BCVA, best corrected visual acuity; CRT, central retinal thickness; DM, differences median; R, Wilcoxon rank effect size.

The results of the analysis with the logistic regression model to examine the effect of individual variables, characterizing the onset of therapy, on the chance of long‐term treatment are shown in Table 5. The model achieved a moderate fit and explains 19.24% of the variation in the probability of long‐term therapy (Likelihood Ratio Test: *p* < 0.001). Females have a 16.7% higher chance of long‐term treatment compared to males, and each additional year of age at the treatment enrolment increases the chance of early treatment failure by 5.3%. Patients who have been treated prior to enrolment in the RTPMR have a 61.3% higher chance of long‐term therapy compared to treatment‐naïve. Regarding clinical outcomes, initial BCVA is less important when BCVA is known after subsequent injections. The BCVA after the third injection has the most significant effect on the chance of long‐term therapy, with an increase of 0.1 logMAR reducing the chance of long‐term therapy by about 9.6%. Subsequent measurements of CRT values show no significant effect on treatment duration, and the first measurement's effect remains negligible. Patients who received ranibizumab at the start of therapy showed a 25.1% higher chance of therapy failure compared to patients treated with aflibercept. The timing of the second and third injections also showed a significant effect, showing that each additional month between the first and second injections reduced the odds of long‐term therapy by 5.2%, and each additional month between the first and third injections increased the odds of long‐term therapy by 7.2%. The favourable increase in time to the third injection may be partly due to the numerical advantage of continued therapies in the long‐term treatment group.

**TABLE 5:** Logistic regression results to model the chance of long‐term treatment based on patient characteristics and early outcomes.

Characteristics	Odds ratio (95% CI)	*p* value
Sex (Male = 0, Female = 1)	1.1673 (1.0761–1.2663)	<0.001
Age, years	0.9470 (0.9421–0.9519)	<0.001
Therapy (New = 0, Continued = 1)	1.6133 (1.4606–1.7821)	<0.001
Medication (Aflibercept = 0, Ranibizumab = 1)	0.7489 (0.6821–0.8224)	<0.001
BCVA, logMAR		
Initial	1.0763 (0.8174–1.4172)	0.600
After first injection	0.6690 (0.4624–0.9679)	0.033
After second injection	0.6072 (0.4227–0.8721)	0.007
After third injection	0.0387 (0.0284–0.0527)	<0.001
CRT, μm		
Initial	0.9992 (0.9989–0.9995)	<0.001
After first injection	0.9995 (0.9990–0.9999)	0.028
After second injection	0.9999 (0.9995–1.0004)	0.802
After third injection	1.0003 (0.9999–1.0006)	0.139
Injection time, months		
Second injection	0.9484 (0.9010–0.9983)	0.043
Third injection	1.0724 (1.0350–1.1112)	<0.001

Abbreviations: BCVA, best corrected visual acuity; CRT, central retinal thickness.

**FIGURE 4:**
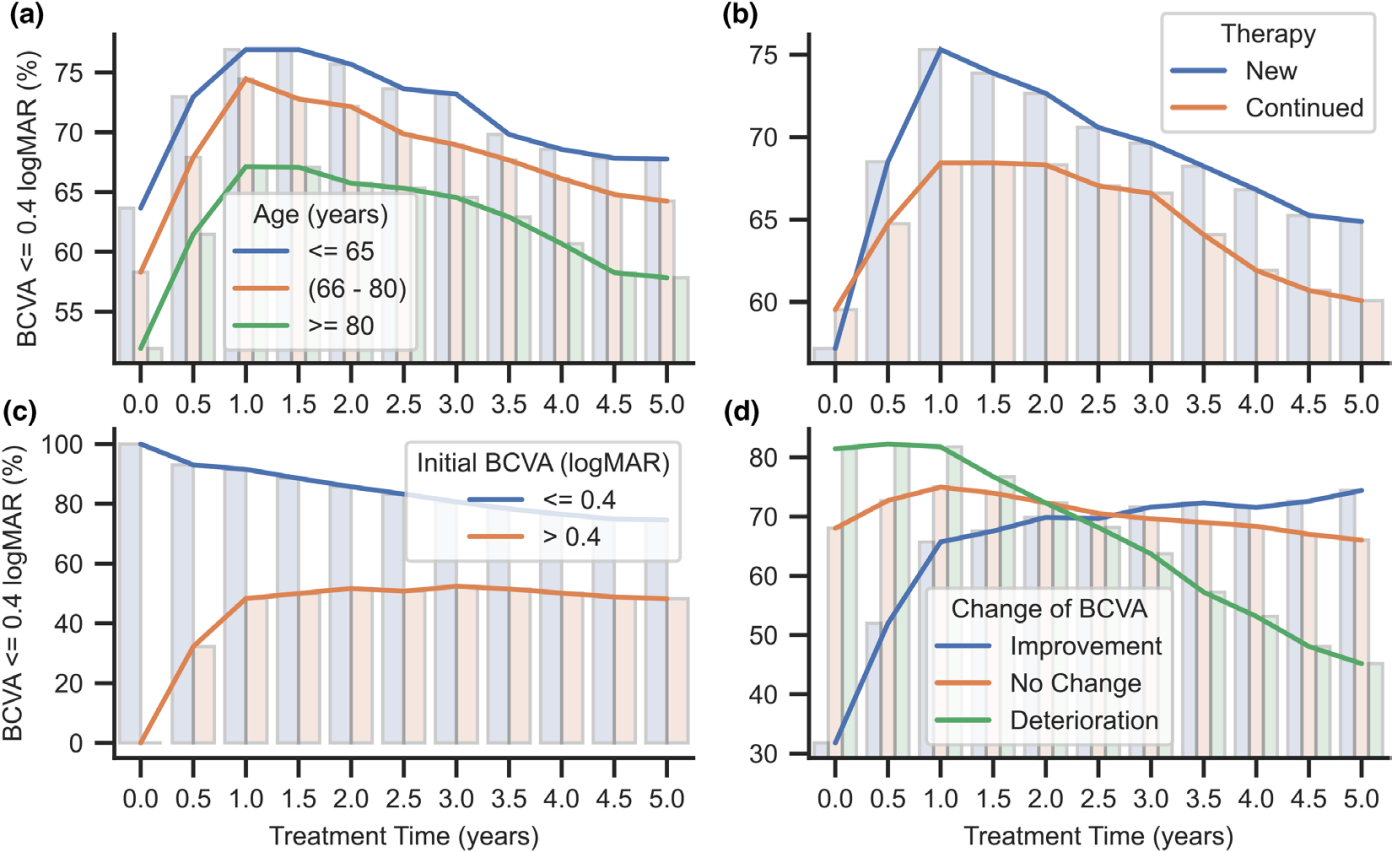
Percentage summaries of the number of eyes with BCVA below or equal to 0.4 logMAR in subsequent years of treatment, divided into groups based on age (a), therapy (b), initial BCVA (c) and change of BCVA after treatment (d).

Of the long‐term‐treated patients, 4879 (63.9%) were women and 2751 (36.1%) were men. The age group between 66 and 80 (at the time of RTPMR inclusion) included 4915 (64.4%) patients, with 1365 (17.8%) patients younger than 66 and 1351 (17.8%) older than 80. A comparison of initial characteristics and outcome measures between the sex and age groups of long‐term treatment patients showed no significant differences. Although there is no significant difference in BCVA between age groups, analysis of the proportion of patients with BCVA no greater than 0.4 logMAR shows that both the initial BCVA and the BCVA achieved and maintained during treatment differ between age groups. At the same time, the effect of age on the ability to obtain improvement and maintain the results is much less observable (Figure 4a).

The therapies of 2224 (29.1%) eyes treated long term were a continuation of the preceding treatment outside the RTPMR. A comparison of the characteristics and outcomes of the previously treated with treatment‐naïve showed no significant differences. In this case, it is particularly interesting that the groups did not differ regarding initial BCVA (Mann–Whitney *U* test: *p* = 0.16) and CRT (Mann–Whitney *U* test: *p* = 0.31). The average time since the last injection in treatment prior to participation in the RTPMR in the long‐term treatment group is 218 days, which may account for the deterioration of visual acuity in this group despite previous treatment. The proportion of BCVA improvements after the first year of new therapies has reached higher than that of continuing therapies. The continued deterioration trend remains similar in both groups (Figure 4b).

The initial BCVA of more than 0.4 logMAR significantly worsens the possibility of achieving better BCVA results throughout long‐term therapy. The BCVA differed significantly after the third injection compared to patients with baseline BCVA ≤0.4 logMAR (Table 6). The greatest improvement in BCVA occurred during the first year of therapy, and the effects persisted further into the course of therapy (Figure 4c). The simultaneous gradual worsening of BCVA in patients seeing better at baseline reduced differences between groups at the last follow‐up point. Comparison of CRT between the groups showed no significant differences at either the start of therapy or at subsequent stages. Patients with an initial BCVA of no more than 0.4 logMAR are significantly younger than patients starting with worse visual acuity. At the same time, there were no significant differences in the values of indicators characterizing the treatment strategy (drugs administered, their changes during therapy and the number of injections) (Table 6).

**TABLE 6:** Comparison of characteristics and outcome measures between patients with different initial visual acuity and different change of visual acuity after observed long‐term treatment in the Retinal Therapeutic Program Monitoring Registry.

Characteristics	Initial BCVA, logMAR			Change of BCVA after observed treatment			
	≤0.4, *n* = 4416	>0.4, *n* = 3215	*p* value	Deterioration, *n* = 2107	No change, *n* = 2605	Improvement, *n* = 2919	*p* value
Age, years, mean ± std	72.37 ± 7.83	73.68 ± 7.67	<0.001^a^, d = −0.17	73.24 ± 7.73	72.74 ± 7.93	72.85 ± 7.70	0.076^e^
Sex, *n* (%)			0.44^b^				0.051^b^
Female	2807 (63.6)	2072 (64.4)		1327 (63.0)	1636 (62.8)	1916 (65.6)	
Male	1609 (36.4)	1143 (35.6)		780 (37.0)	969 (37.2)	1003 (34.4)	
BCVA, logMAR, median (quartile deviation)							
Initial	‐	‐	‐	0.30 (0.10)	0.30 (0.16)	0.52 (0.15)	<0.001^d^, *ε* ^2^ = 0.284
After third injection	0.22 (0.10)	0.49 (0.15)	<0.001^c^, rg = 0.68	0.30 (0.12)	0.30 (0.17)	0.40 (0.20)	<0.001^d^, *ε* ^2^ = 0.062
Last measured	0.30 (0.17)	0.49 (0.20)	<0.001^c^, rg = 0.33	0.60 (0.15)	0.30 (0.14)	0.30 (0.12)	<0.001^d^, *ε* ^2^ = 0.275
Mean	0.28 (0.09)	0.46 (0.12)	<0.001^c^, rg = 0.58	0.40 (0.10)	0.32 (0.14)	0.34 (0.11)	<0.001^d^, *ε* ^2^ = 0.028
CRT, μm, median (quartile deviation)							
Initial	301.0 (51.9)	315.0 (53.5)	0.088^c^	303.0 (52.5)	306.0 (53.0)	310.0 (51.0)	0.018^d^, *ε* ^2^ = 0.001
After third injection	276.0 (49.8)	275.0 (48.0)	0.76^c^	280.0 (49.6)	277.0 (47.5)	271.5 (49.0)	0.002^d^, *ε* ^2^ = 0.002
Last measured	248.0 (40.5)	248.0 (44.5)	0.52^c^	253.0 (47.5)	250.0 (43.5)	245.0 (38.5)	0.002^d^, *ε* ^2^ = 0.002
Mean	266.6 (33.5)	264.0 (34.3)	0.036^c^, rg = −0.03	271.4 (35.0)	264.9 (34.2)	261.7 (32.4)	<0.001^d^, *ε* ^2^ = 0.007
Therapy, *n* (%)			0.06^b^				<0.001^b^, *V* = 0.06
New	3092 (70.0)	2315 (72.0)		1419 (67.4)	1828 (70.2)	2160 (74.0)	
Continued	1324 (30.0)	900 (28.0)		688 (32.6)	777 (29.8)	759 (26.0)	
First medication, *n* (%)			<0.001^b^, *V* = 0.04				<0.001^b^, *V* = 0.07
Aflibercept	3418 (77.4)	2608 (81.1)		1645 (78.1)	1975 (75.8)	2406 (82.4)	
Ranibizumab	998 (22.6)	607 (18.9)		462 (21.9)	630 (24.2)	513 (17.6)	
Medication change, *n* (%)			0.033^b^, *V* = 0.02				<0.001^b^, *V* = 0.08
Unchanged	2414 (54.7)	1837 (57.1)		1046 (49.6)	1463 (56.2)	1742 (59.7)	
Changed	2002 (45.3)	1378 (42.9)		1061 (50.4)	1142 (43.8)	1177 (40.3)	
Number of injections per year, *n*, median (quartile deviation)	5.49 (1.08)	5.37 (1.05)	<0.001^c^, rg = −0.04	5.52 (1.08)	5.44 (1.05)	5.36 (1.05)	0.002^d^, *ε* ^2^ = 0.001

Abbreviations: BCVA, best corrected visual acuity; CRT, central retinal thickness; D, Cohen's d; rg, rank‐biserial correlation; *V*, Cramer's *V*; *ε* ^2^, Kruskal‐Wallis effect size; *η* ^2^, ANOVA effect size.

^a^ Welch *t*‐test.

^b^ Chi‐square test.

^c^ Mann–Whitney *U* test.

^d^ Kruskal–Wallis test.

^e^ ANOVA.

In the division by change of BCVA after observed treatment, significant differences are found in the initial and last measured BCVA (Table 6). In the case of worse BCVA, significant improvement is observed already after the third injection (Wilcoxon signed‐rank test, initial BCVA vs BCVA after third injection: *p* < 0.001, *r* = 0.65) and continued in further treatment (Wilcoxon signed‐rank test, BCVA after third injection vs last measured BCVA: *p* < 0.001, *r* = 0.61). In the group in which deterioration of BCVA was observed, a significant change occurred only between the third injection and the last measurement (Wilcoxon signed‐rank test, initial BCVA vs BCVA after third injection: *p* < 0.001, *r* = 0.81). A non‐significant change in BCVA at the beginning of treatment occurred in both the deterioration and no change groups, with relatively similar levels of initial BCVA (Figure 4d). The proportion of patients with BCVA of no more than 0.4 logMAR in the improving group increases sharply during the first year of therapy. The increase continues after this period, while the other groups encounter a decrease in the proportion with different dynamics. After the second year of therapy, a characteristic moment of equalizing proportions in all groups can be observed (Figure 4d).

**FIGURE 5:**
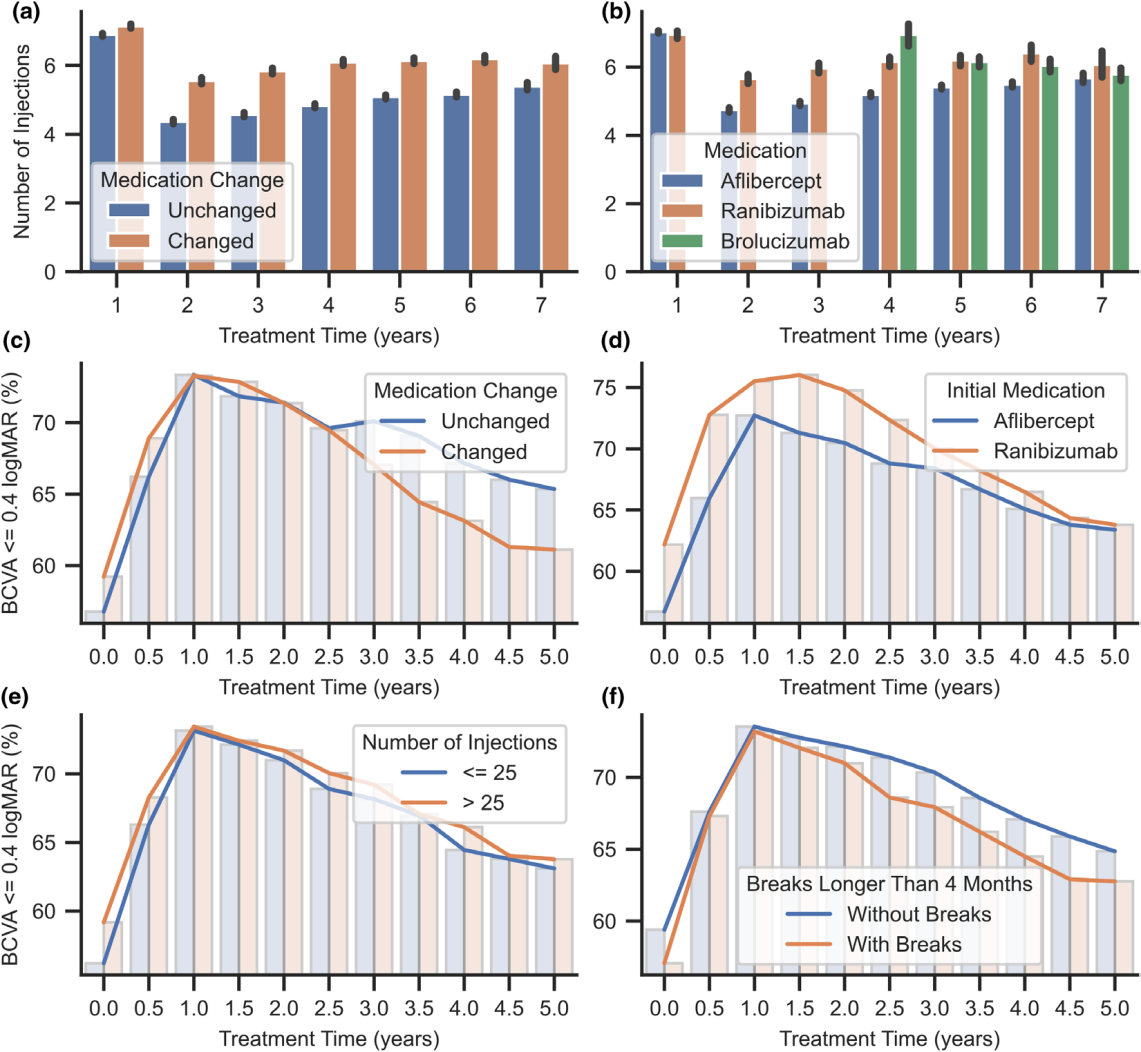
Mean number of injections in subsequent years of treatment according to medication change (a) and current medication in the therapy (b), and percentage summaries of the number of eyes with BCVA below or equal to 0.4 logMAR in subsequent years of treatment, divided into groups based on medication change (c), initial medication (d), number of injections (e) and breaks in the therapy (f).

The average number of injections is highest in the first year of therapy, much lower in the second, and gradually increases in subsequent years (Figure 5a). From the second year of therapy onwards, the observed average number of injections is higher in patients who changed medications throughout long‐term therapy compared to patients treated continuously with the same drug. The average number of injections of ranibizumab is higher than that of aflibercept during this period (Figure 5b). Ranibizumab was provided as the first drug in 1605 (21.0%) cases. At the same time, the drug was changed significantly more often than aflibercept (Chi‐Square Test: *p* < 0.001, *V* = 0.36). Finally, the entire long‐term ranibizumab therapy was used in only 337 (4.4%) patients. Despite the lack of significant differences in initial BCVA between drugs, among ranibizumab‐treated patients, about 5% more achieved an initial BCVA of less than 0.4 logMAR. This difference persists during the first years of therapy and gradually reduces thereafter (Figure 5d). The BCVA and CRT results obtained over the course of therapy show no difference between the drugs used and the number of injections per year is slightly higher with ranibizumab (Mann–Whitney *U* test: *p* < 0.001, rg = 0.1).

In the entire study group of patients on long‐term therapy, 3380 (44.3%) patients had at least one drug change during therapy. A comparison of patients' ages at the start of therapy showed that drug changes were made in the significantly younger group (Welch *t*‐test: *p* < 0.001, d = 0.26). This group showed a slightly higher CRT after the third injection relative to the group without drug changes (Mann–Whitney *U*‐test: *p* < 0.001, rg = 0.11). About BCVA, no significant differences were observed, but the percentage analysis shown in Figure 5c indicates a more downward trend in the percentage of better‐seeing patients after 2.5 years of therapy. At the same time, the group with the drug change showed a significantly higher number of injections per year (Mann–Whitney *U* test: *p* < 0.001, rg = 0.33).

Considering the number of injections in the first 5 years of long‐term treatment, the number did not exceed 25 injections for 3347 (43.9%) patients. Compared to the group with a higher number of injections, the age of these patients is significantly higher (Table 7). Significant differences were also shown for CRT after the third injection and at the end of the observation period. The results also confirm previous observations of a significantly higher proportion of patients with a change of drug during therapy in the group with a higher number of injections (Table 7). Analysis of the proportion of patients with a BCVA of no more than 0.4 logMAR throughout therapy showed no difference between groups (Figure 5e).

**TABLE 7:** Comparison of characteristics and outcome measures between patients with different number of injections and according to breaks in the observed long‐term treatment in the Retinal Therapeutic Program Monitoring Registry.

Characteristic	Number of injections			Breaks longer than 4 months		
	≤25, *n* = 3380	>25, *n* = 4284	*p* value	Without breaks, *n* = 2489	With breaks, *n* = 5142	*p* value
Age, years, mean ± std	73.76 ± 7.64	72.27 ± 7.84	<0.001^a^, d = 0.19	72.24 ± 7.76	73.25 ± 7.78	<0.001^a^, d = 0.13
Sex, *n* (%)			<0.001^b^, *V* = 0.05			0.002^b^, *V* = 0.04
Female	2237 (66.8)	2642 (61.7)		1531 (61.51)	3348 (65.11)	
Male	1110 (33.2)	1642 (38.3)		958 (38.49)	1794 (34.89)	
BCVA, logMAR, median (quartile deviation)						
Initial	0.40 (0.24)	0.40 (0.19)	<0.001^c^, rg = −0.05	0.40 (0.19)	0.40 (0.24)	0.005^c^, rg = 0.04
After third injection	0.30 (0.16)	0.30 (0.14)	0.12^c^	0.30 (0.14)	0.30 (0.16)	0.21^c^
Last measured	0.40 (0.14)	0.40 (0.14)	0.72^c^	0.40 (0.14)	0.40 (0.20)	0.032^c^, rg = 0.03
Mean	0.34 (0.12)	0.34 (0.12)	0.92^c^	0.34 (0.12)	0.35 (0.12)	0.008^c^, rg = 0.04
CRT, μm, median (quartile deviation)						
Initial	305.0 (52.5)	309.0 (53.5)	0.50^c^	309.0 (53.2)	306.0 (52.0)	0.36^c^
After third injection	267.0 (46.5)	284.0 (50.0)	<0.001^c^, rg = 0.14	286.0 (50.5)	271.5 (48.0)	<0.001^c^, rg = −0.12
Last measured	249.0 (41.5)	259.0 (46.0)	<0.001^c^, rg = 0.11	251.0 (43.0)	247.0 (41.5)	<0.001^c^, rg = −0.05
Mean	261.3 (34.5)	275.0 (36.8)	<0.001^c^, rg = 0.16	271.6 (34.9)	262.7 (33.5)	<0.001^c^, rg = −0.11
Therapy, *n* (%)			<0.001^b^, *V* = 0.09			0.59^b^
New	2224 (66.5)	3183 (74.3)		1774 (71.27)	3633 (70.65)	
Continued	1123 (33.5)	1101 (25.7)		715 (28.73)	1509 (29.35)	
First medication, *n* (%)			<0.001^b^, *V* = 0.07			0.022^b^, *V* = 0.03
Aflibercept	2759 (82.4)	3267 (76.2)		1927 (77.42)	4099 (79.72)	
Ranibizumab	588 (17.6)	1017 (23.7)		562 (22.58)	1043 (20.28)	
Medication change, *n* (%)			<0.001^b^, *V* = 0.24			<0.001^b^, *V* = 0.13
Unchanged	2335 (69.76)	1916 (44.7)		1159 (46.56)	3092 (60.13)	
Changed	1012 (30.24)	2368 (55.2)		1330 (53.44)	2050 (39.87)	
Number of injections per year, *n*, median (quartile deviation)	‐	‐	‐	6.66 (0.74)	4.75 (0.86)	<0.001^c^, rg = −0.77

Abbreviations: BCVA, best corrected visual acuity; CRT, central retinal thickness; d, Cohen's d; rg, rank‐biserial correlation; *V*, Cramer's *V*.

^a^ Welch *t*‐test.

^b^ Chi‐square test.

^c^ Mann–Whitney *U* test.

Breaks more than 4 months between the injections occurred in significantly older patients (Table 7). It also shows a significant difference in the proportion of drug changes during therapy—patients who did not interrupt therapy had significantly more drug changes. At the same time, this group is characterized by a significantly higher number of injections than the group of patients with treatment interruptions. The results obtained during therapy show no variation within BCVA, while the CRT of patients with interruptions of therapy is significantly lower after three injections, and the mean value is lower compared to patients with continuity of treatment. In addition, this phenomenon occurs despite the lack of differences in CRT at the beginning of therapy (Table 7). A comparison of the proportion of patients with BCVA no greater than 0.4 logMAR shows a slightly greater downward trend after the first year of therapy for patients with interruptions. However, this effect is insignificant, as the difference between groups is less than 3% at the last observed follow‐up point (Figure 5f).

**FIGURE 6:**
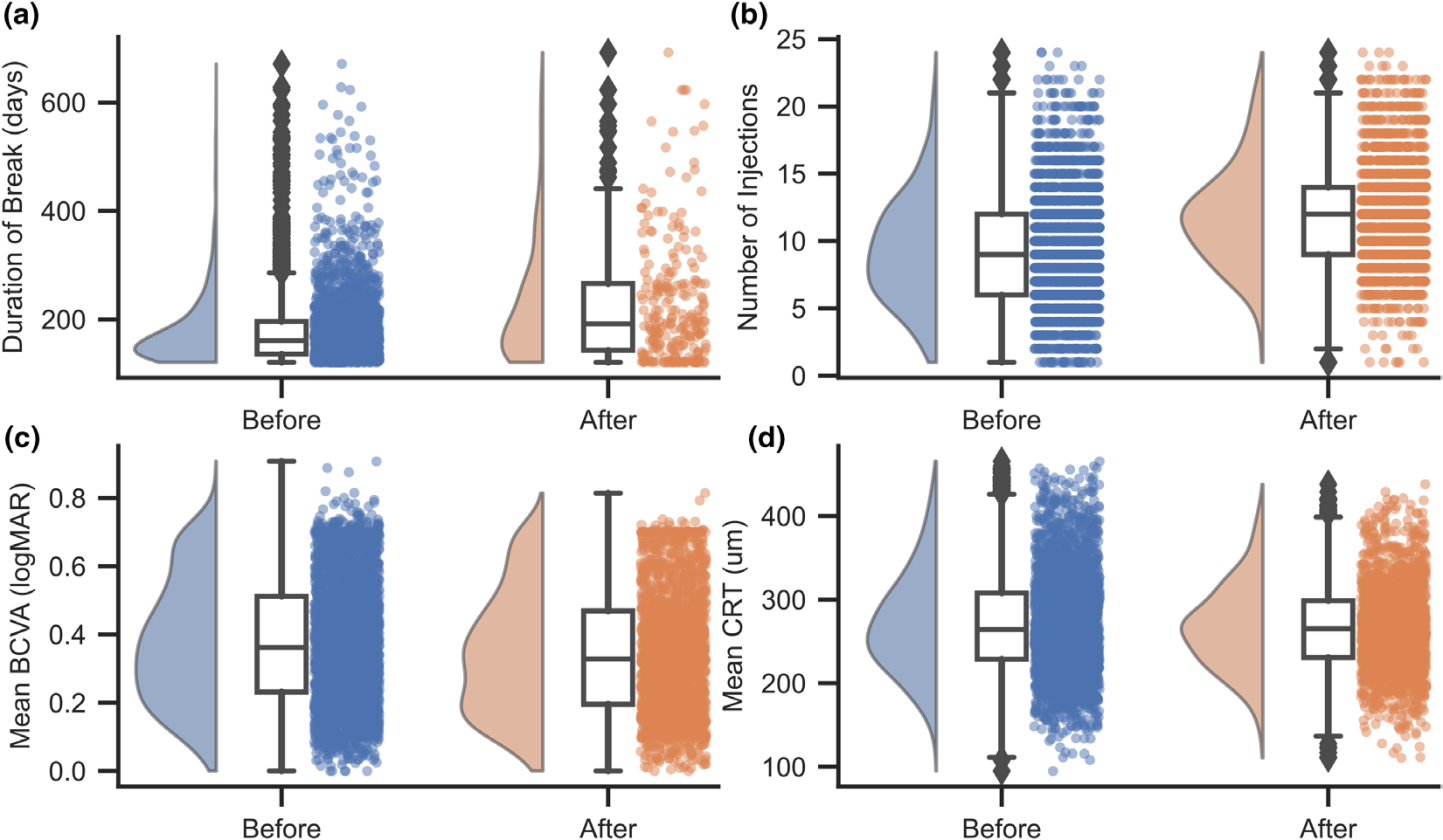
Distributions of treatment break duration (a), number of injections over 2 years of follow‐up (b), mean visual acuity (c) and mean central retinal thickness (d) between homogeneous treatment groups before and after the introduction of the criterion to terminate treatment episode after a gap of more than 4 months.

A comparative analysis of homogeneous treatment groups before and after the introduction of the criterion to exclude patients after a gap of more than 4 months (with the possibility of requalification, if necessary) showed that the number of breaks between injections longer than 4 months dropped significantly after the introduction of the criterion. In the treatment group before its introduction, it averaged 0.93 breaks per patient, and after, only 0.14 breaks per patient. At the same time, the median length of breaks of 192 days is significantly higher in the group after introducing the criterion, relative to 161 days before (Mann–Whitney *U* test: *p* < 0.001, rg = 0.23). This means a significant reduction in the number of interruptions, slightly longer than 4 months, as a result of the criterion (Figure 6a). The number of injections given during the analysed 2‐year treatment period for both groups increased significantly after the introduction of the criterion (Mann–Whitney *U* test: *p* < 0.001, rg = 0.37) (Figure 6b). The mean visual acuity in both groups is at similar levels, with a slightly lower median value in the group after the introduction of the criterion (Mann–Whitney *U* test: *p* < 0.001, rg = 0.1) (Figure 6c), while no significant differences were found for the mean CRT (Mann–Whitney *U* test: *p* = 0.14) (Figure 6d).

## 4 DISCUSSION

Unlike traditional national registries that primarily document real‐world treatment patterns, the RTPMR functions as a structured, system‐driven framework that not only records treatment data but also enforces adherence to standardized therapeutic protocols. This programme, managed by the Coordinating Committee (CC) on behalf of the national health insurance provider, serves as a regulatory mechanism ensuring compliance with evidence‐based guidelines, rather than merely reflecting physician‐driven clinical decision‐making. Before the introduction of the RTPMR in Poland at the end of 2015, reimbursement was based on hospital procedure billing, rather than a structured national programme. There was no external oversight, and treatment was often irregular and dependent on the individual hospital's contract with the national health insurer. This variability led to inconsistencies in access to therapy and continuity of care. Currently, patients who do not meet the eligibility criteria still receive treatment with reimbursed bevacizumab, which is also administered without the same level of strict monitoring as the therapies within the RTPMR.

A key distinction of the Polish system is its role in standardizing treatment nationwide, minimizing disparities in therapeutic access and quality across different healthcare centres. By incorporating rigorous monitoring and control mechanisms, the programme prevents deviations from best clinical practices and optimizes resource allocation by avoiding unnecessary or ineffective treatments. All major treatment decisions—particularly patient eligibility for therapy—must be verified by an expert in the field, ensuring that only those meeting well‐defined criteria receive anti‐VEGF treatment. This stringent oversight contrasts with registries like the IRIS Registry or the Fight Retinal Blindness! Registry, which primarily aggregate observational data without directly influencing treatment protocols. Comprehensive expert oversight also enables the inclusion of smaller local centres and ensures broader access to treatment. It aligns with the principle of decentralizing healthcare services to improve treatment availability and equity.

Prior authorization by CC is conducted online with an average response time of 1–2 days. This system prevents unnecessary treatments and irrational resource allocation, while simultaneously providing both patients and physicians with confirmation of the appropriateness of the therapy. The RTPMR also optimizes drug utilization, negotiating directly with pharmaceutical companies, unlike the more insurance‐dependent IRIS Registry, where economic disparities influence treatment continuity. Furthermore, by integrating cost‐effectiveness considerations with clinical decision‐making, this approach ensures that healthcare resources are allocated efficiently, maximizing both patient outcomes and economic sustainability. Consequently, the RTPMR does not merely describe clinical practice but actively shapes and refines it, establishing a benchmark for structured, accountable, and standardized care delivery in ophthalmology.

The RTPMR confirms a higher prevalence of nAMD in females (62.99%), consistent with global epidemiological trends. Similarly, data from the IRIS Registry (USA), Fight Retinal Blindness! Registry (Australia and New Zealand), and Swedish Macula Register report a predominance of female patients, with proportions ranging from 55% to 65% (Debourdeau et al., 2024; Westborg et al., 2017; Wykoff et al., 2024). This aligns with the established understanding that females generally live longer, increasing their exposure to age‐related diseases. Additionally, hormonal and genetic factors may contribute to the increased susceptibility of females to nAMD, as the age‐gender distribution of the general population indicates that life expectancy is only a partial cause of the difference in the gender distribution of nAMD.

A notable discrepancy arises in the RTPMR regarding the age of treatment initiation, with males requiring anti‐VEGF therapy approximately 2 years earlier than females. This trend is less pronounced in other registries, where gender differences in initiation age are generally minimal (Rudnicka et al., 2012). One potential explanation could be variations in healthcare‐seeking behaviour, with men being diagnosed earlier due to a higher likelihood of undergoing routine medical check‐ups in Poland, or due to a more aggressive disease phenotype in males. Research on the effect of oestrogen and other hormonal factors on nAMD progression remains inconclusive, with some suggesting protective roles of hormones and others showing no significant effect. Variations in smoking rates, dietary patterns and other lifestyle factors between men and women might also influence nAMD onset and severity (Thornton et al., 2005). Additionally, shorter life expectancy for Polish males may contribute to an apparent earlier treatment initiation, as fewer older men remain in the patient pool compared to women. This could artificially inflate the observed difference in treatment initiation age.

The analysis of long‐term treatment success versus failure in Poland reveals that younger patients have a significantly higher probability of maintaining therapy over 5 years. Comparing this to the Fight Retinal Blindness! Registry, which also reports worse visual outcomes in older populations, it appears that Poland follows a similar trend (Gillies et al., 2020). However, the extent of the negative impact of age is more pronounced in the Polish dataset, possibly reflecting differences in treatment adherence, access to care or other systemic factors. Odds of nAMD treatment nonpersistence in the IRIS Registry also were greatest in the elderly (Khurana et al., 2023).

The RTPMR also confirms a higher gain in visual acuity in patients who continue treatment long term, suggesting that early functional improvements in vision are key predictors of adherence. This pattern is in line with the Swedish Macula Register, which found that patients who experience initial vision gains are more likely to remain in therapy (Westborg et al., 2017). However, the Polish data contrast with IRIS Registry findings, where systemic factors (insurance coverage, economic disparities) play a more dominant role in determining treatment adherence, rather than initial BCVA improvement alone (Khurana et al., 2023).

The study underscores the critical role of early visual acuity response after initial injections in determining long‐term treatment retention. This aligns with previous findings (e.g. CATT, IVAN and Fight Retinal Blindness! registries) demonstrating that initial BCVA gains correlate with sustained visual function over time (Chakravarthy et al., 2013; Ying et al., 2015). Early non‐responders were more prevalent in the ranibizumab cohort, suggesting that aflibercept's pharmacokinetics may offer superior early disease control, leading to better functional outcomes over time. This underscores the importance of aggressive early treatment strategies to maximize functional recovery and prevent long‐term deterioration, a principle supported by other real‐world analyses. The Polish dataset reveals that a subset of patients with similar anatomical improvements in CRT failed to achieve meaningful BCVA gains, ultimately leading to early treatment discontinuation. It may indicate the influence of another morphological change, like ellipsoid zone disruption or external limiting membrane integrity (Metrangolo et al., 2021).

Patients with better baseline BCVA lower or equal to 0.4 logMAR were more likely to remain in long‐term treatment and preserve better vision (Ho et al., 2020). However, the relationship between initial BCVA and treatment adherence is complex and multifaceted (Ramakrishnan et al., 2020). Patients presenting with lower baseline BCVA often exhibit more substantial relative improvements after initiating anti‐VEGF therapy. This is attributed to the greater potential for vision improvement in eyes with poorer initial BCVA. For instance, a study analysing data from the Comparison of Age‐Related Macular Degeneration Treatments Trials (CATT) found that individuals with worse baseline BCVA achieved more significant letter score gains compared to those with better baseline BCVA (Ying et al., 2015). Despite experiencing greater initial improvement at the start of treatment, patients with lower baseline visual acuity do not achieve the same level of final visual acuity as those who had better initial vision and, consequently, a smaller overall gain. This effect is mirroring data from, for example, Swedish Macula Register (Lövestam Adrian et al., 2019).

Longer gaps between the first and second injections negatively impacted long‐term retention, whereas a slightly extended interval between the second and third injections correlated with better treatment persistence. This nuanced finding diverges from prior real‐world studies, which typically associate early intensive loading‐phase dosing with superior retention and functional outcomes. Although it is not certain, it is reasonable to assume that ophthalmologists tended to slightly increase the interval after the second injection in patients who did not have retinal fluid. It is also possible that this group included patients who were incorrectly reported as new therapies when in fact they were continuing treatment.

The introduction of a 4‐month inactivity termination rule in November 2020 had a profound impact on treatment retention. The rule significantly reduced treatment interruptions, potentially reinforcing adherence. A portion of patients may have experienced prolonged treatment gaps exceeding 4 months before being requalified, contributing to extended discontinuation periods. The requirement for requalification following prolonged gaps may have discouraged reinstatement, inadvertently increasing long‐term dropout rates. However, the anticipated need for requalification, along with administrative procedures and the requirement for approval from the CC, served as an additional incentive for physicians to take measures to ensure that patients remained in the RTPMR.

The RTPMR data indicate that aflibercept is associated with superior long‐term visual retention compared to ranibizumab, as evidenced by higher proportions of patients maintaining functional vision and lower discontinuation rates due to disease progression. These findings align with large‐scale real‐world studies, such as the Fight Retinal Blindness! registry and the Swedish Macula Register, both reporting greater long‐term visual stability with aflibercept. While randomized controlled trials (e.g. VIEW 1/2) demonstrated similar 1‐year BCVA gains for both drugs, real‐world aflibercept‐treated patients had a significantly higher likelihood of maintaining vision over extended follow‐up periods (Park et al., 2017). Additionally, disparities in treatment adherence due to socio‐economic factors and healthcare access may influence long‐term treatment efficacy, making registry‐based insights invaluable for refining management strategies.

The need for fewer injections per year in aflibercept‐treated patients compared to ranibizumab suggests a lower treatment burden, which may contribute to improved adherence. This aligns with global registry findings, where proactive T&E regimens with aflibercept have led to better patient retention and fewer missed treatments (Debourdeau et al., 2024). The structured treatment approach in Poland, with its standardized therapeutic programme, further supports adherence by ensuring systematic follow‐ups. Additional factors influencing adherence include patient education, transportation barriers and treatment expectations, which may impact outcomes beyond the pharmacologic properties of the drugs themselves.

Although the presented results are based on a detailed analysis of a large dataset comprising most patients treated for nAMD in Poland, several limitations should be considered when interpreting the findings. The visual acuity measurements may vary due to the different assessment methods between centres (Snellen and ETDRS charts) and the fact that measurements were performed in routine clinical settings. Similarly, differences in OCT devices across centres and variability in CRT measurement algorithms may have introduced additional heterogeneity. Moreover, despite attempts to account for all relevant factors in our analysis, evolving inclusion criteria and additional treatment termination rules in the RTPMR during the observation period may have introduced residual, difficult‐to‐control bias. Finally, the specific conditions of the national healthcare system and treatment framework within the RTPMR may limit the generalizability of our study outcomes. Consequently, treatment adherence and visit frequency may not fully reflect those observed in healthcare systems without full reimbursement or with different monitoring schedules. Therefore, clinical outcomes and reasons for treatment discontinuation might differ in other settings.

## 5 CONCLUSION

Medical registries provide essential insights into the long‐term efficacy, adherence and policy impact of nAMD treatments. Early visual acuity response, therapeutic choice and systemic treatment adjustments critically influence sustained outcomes and patient adherence. Although systemic improvements enhanced treatment continuity and injection frequency, they did not translate into better visual results, underscoring the importance of individualized management. Future research should prioritize harmonizing international registry methodologies to enable robust cross‐country comparisons and support the development of globally applicable treatment guidelines.

## CONFLICT OF INTEREST STATEMENT

The authors declare that they have no competing interests.

## Data Availability

The datasets generated and analysed during the current study are available from the corresponding author on reasonable request.
